# Advanced Practitioners Are Not Mid-Level Providers

**Published:** 2012-09-01

**Authors:** Catherine S. Bishop

**Affiliations:** Dr. Bishop is a Doctor of Nursing Practice and Oncology Nurse Practitioner at Sibley Memorial Hospital, Johns Hopkins Medicine, in Washington, DC.

While I was completing my nurse practitioner program, I interviewed for a PRN position at a large private oncology practice. The oncologist with whom I spoke was aware that I was continuing my graduate nursing education; she spoke of how they might consider me for employment as an oncology nurse practitioner upon my graduation. As she talked about her current staff, she used a descriptor I had never heard before: mid-level. I truly did not know what she meant, or even who she was speaking about. However, I soon realized she was talking about the nurse practitioners (NPs) and physician assistants (PAs) who were part of her staff.

I was puzzled. Was the term "mid-level" a way of quantifying the amount of care the NPs and PAs provided? Was it a way of qualifying the type of care they provided? I felt confused and belittled. I was in the midst of graduate level (advanced) education and as such would be practicing at an advanced level of nursing. The scope of practice within my home state allowed me to practice medicine under the supervision of a collaborating physician. I was dismayed at the term "mid-level" and what it must sound like to a patient, a family member, or another health-care professional. I did not seek employment at that practice upon graduation.

## A Governmental Identifier

The term "mid-level practitioner" is used by the US Department of Justice’s Drug Enforcement Administration (DEA) to identify a group of health-care individuals for the purpose of monitoring controlled substances. According to the website of the DEA, Office of Diversion Control, "Pursuant to Title 21, Code of Federal Regulations, Section 1300.01(b28), the term mid-level practitioner means an individual practitioner, other than a physician, dentist, veterinarian, or podiatrist, who is licensed, registered, or otherwise permitted by the United States or the jurisdiction in which he/she practices, to dispense a controlled substance in the course of professional practice. Examples of mid-level practitioners include, but are not limited to, health-care providers such as nurse practitioners, nurse midwives, nurse anesthetists, clinical nurse specialists and physician assistants who are authorized to dispense controlled substances by the state in which they practice." Medicare uses the term "non-physician practitioner" to describe advanced practice nurses (APNs) and PAs.

## Taking Control

Although it is not my intention to fight the federal government regarding the terms it uses to describe both APNs and PAs, it is worthwhile to attempt to educate the medical community regarding appropriate titles for health-care practitioners who have advanced degrees. The title or term "mid-level" is certainly not appropriate. Virtually all APNs (NPs, clinical nurse specialists, nurse anesthetists, and nurse midwives) have at least a master’s degree and many hold doctorates. We are clinicians, educators, and researchers who are highly trained to care for and manage patients with a variety of illnesses. Within oncology, we have specialized training, certification, and education that allow us to work alongside our physician partners. We collaborate with members of many disciplines within medicine and nursing and provide unique care to all that we serve. All PAs are educated in master’s degree programs and trained in the medical model designed to harmonize with their supervising physician’s practice. Additionally, there is a clinical doctorate in emergency medicine for PAs offered through Brooke Army Medical Center (Salyer, 2008).

There is nothing "mid" about either an APN or a PA. I think all would agree that we provide a high level of care. Our skill set, education, training, and knowledge go above and beyond what would be considered mid-level. I propose that we be in charge of what we are called, not the government or other entities. Respectfully educating our institutions, human resource departments, recruiters, and medical colleagues is a first step in changing the terminology.

## Progress

Some institutions and organizations have created appropriate titling for NPs and PAs. Dartmouth Hitchcock Medical Center refers to both NPs and PAs as "associate providers." Both NPs and PAs within this institution frequently hold faculty appointments at the Geisel School of Medicine at the instructor level. The American Society of Clinical Oncology (ASCO) has always embraced the role of the NP and the PA. They have been instrumental in finding ways to better assimilate advanced practitioners into oncology practices with the goal of improving patient care and increasing provider satisfaction (Towle et al., 2011). ASCO has partnered with the Oncology Nursing Society on many common issues. Our collective hope is to continue this great partnership and replace the term "non-physician practitioner" with "advanced practitioner," which is more in keeping with our experience and education.

## Conclusion

I believe that all APNs and PAs would echo the sentiments of our NP colleague Alison Moriarty Daley, MSN, APRN, PNP, who stated "There are too many people who need high-quality, dedicated providers; we are such providers and deserve the appropriate respect, recognition, and support from the healthcare community" (2011). Let us stand together and request that the adopted terminology of mid-level, physician extender, and non-physician practitioner be abolished. If we are referred to as a group, call us advanced practitioners. Otherwise, let us simply be called what we are: nurse practitioners and physician assistants.
